# Genetic Basis of Antigenic Variation of SAT3 Foot-And-Mouth Disease Viruses in Southern Africa

**DOI:** 10.3389/fvets.2020.00568

**Published:** 2020-09-08

**Authors:** Lorens Maake, William T. Harvey, Lia Rotherham, Pamela Opperman, Jacques Theron, Richard Reeve, Francois F. Maree

**Affiliations:** ^1^Vaccine and Diagnostic Development Programme, Onderstepoort Veterinary Institute, Agricultural Research Council, Pretoria, South Africa; ^2^Department of Biochemistry, Genetics and Microbiology, Faculty of Agricultural and Natural Sciences, University of Pretoria, Pretoria, South Africa; ^3^Boyd Orr Centre for Population and Ecosystem Health, Institute of Biodiversity, Animal Health and Comparative Medicine, College of Medical, Veterinary and Life Sciences, University of Glasgow, Glasgow, United Kingdom; ^4^Department of Animal Production Studies, Faculty of Veterinary Science, University of Pretoria, Pretoria, South Africa

**Keywords:** foot-and-mouth disease (FMD), Southern African Territory (SAT) type 3, antigenic, cross-reactivity, antigenic matching, phylogeny, virus neutralization test

## Abstract

Foot-and-mouth disease (FMD) continues to be a major burden for livestock owners in endemic countries and a continuous threat to FMD-free countries. The epidemiology and control of FMD in Africa is complicated by the presence of five clinically indistinguishable serotypes. Of these the Southern African Territories (SAT) type 3 has received limited attention, likely due to its restricted distribution and it being less frequently detected. We investigated the intratypic genetic variation of the complete P1 capsid-coding region of 22 SAT3 viruses and confirmed the geographical distribution of five of the six SAT3 topotypes. The antigenic cross-reactivity of 12 SAT3 viruses against reference antisera was assessed by performing virus neutralization assays and calculating the r_1_-values, which is a ratio of the heterologous neutralizing titer to the homologous neutralizing titer. Interestingly, cross-reactivity between the SAT3 reference antisera and many SAT3 viruses was notably high (r_1_-values >0.3). Moreover, some of the SAT3 viruses reacted more strongly to the reference sera compared to the homologous virus (r_1_-values >1). An increase in the avidity of the reference antisera to the heterologous viruses could explain some of the higher neutralization titers observed. Subsequently, we used the antigenic variability data and corresponding genetic and structural data to predict naturally occurring amino acid positions that correlate with antigenic changes. We identified four unique residues within the VP1, VP2, and VP3 proteins, associated with a change in cross-reactivity, with two sites that change simultaneously. The analysis of antigenic variation in the context of sequence differences is critical for both surveillance-informed selection of effective vaccines and the rational design of vaccine antigens tailored for specific geographic localities, using reverse genetics.

## Highlights

- Phylogenetic relationships of the capsid-coding region of SAT3 viruses confirmed the geographical distribution of the southern African topotypes.
- Cross-reactivity between SAT3 reference antisera and SAT3 viruses is notably high.
- Avidity could explain some of the higher cross-reactivity observed.
- Unique amino acid residues may be associated with a change in cross-reactivity.

## Introduction

Foot-and-mouth disease (FMD) continues to be a major burden for livestock owners in endemic countries (1). The occurrence of FMD negatively impacts on the livelihoods of local farmers due to its effects on productivity, food insecurity and losses of income, but also have damaging consequences on international trade in livestock and animal products. The disease is widely distributed in Africa, Asia, and South America where FMD is regarded as endemic. FMD outbreaks particularly affect vulnerable individuals, such as women and children since ~75% of livestock in Africa are raised under the communal smallholder, communal-grazing or pastoral systems that sustain livelihoods of these groups (2, 3). Controlling FMD at its source is therefore a shared interest between endemic and free countries (4). The epidemiology of FMD in sub-Saharan Africa is unique due to the presence of the South African Territories (SAT) serotypes that are almost exclusively endemic, and its continuous maintenance in wildlife (5–7). Therefore, FMD control in livestock is dependent, in part, on an understanding of pathogenesis, persistence, and transmission from African buffalo (*Syncerus caffer*) (7).

Clinically indistinguishable FMD viruses (FMDV) belonging to the SAT serotypes are maintained in buffalo, but differ from each other with respect to their geographic distribution, incidence, outer capsid-coding sequence and antigenicity. SAT2 is the most widely distributed in Africa and is also the serotype most often associated with outbreaks in cattle in southern Africa, followed by SAT1 and then SAT3 (8–10). However, viruses of the SAT1 serotype is most frequently isolated from buffalo (7, 11). Viruses belonging to the SAT3 serotype have the most restricted distribution and essentially occur in southern Africa and in the south-western region of Uganda (12, 13). The SAT3 serotype is also less frequently detected in African buffalo (12). In South Africa, in the Kruger National Park, a SAT3 outbreak occurred in 1958/59 where it involved wildebeest (*Connochaetes taurinus*), kudu (*Tragelaphus strepsiceros*) and sable antelope (*Hippo tragus niger*) (14) and was also detected in Mozambique. Other outbreaks were detected in cattle in Limpopo (Giyani) during 1979/80 (15), in Phalaborwa during 2002 affecting buffalo, in Thulamela during 2006 and in the Kruger National Park (Pafuri) during 2008 affecting impala (*Aepyceros melampus*) as well. The 1979/80 outbreak in Giyani lasted for 9 months and was the longest SAT3 outbreak reported to date (9). Neighboring southern African countries also experienced SAT3 outbreaks during similar times with the most recent outbreaks reported in livestock in Namibia in 2011, Zimbabwe in 1999 and 2013, Zambia in 2015 and 2017, and Mozambique in 2016–2017 (Records of the OIE).

Phylogenetic reconstruction of the partial VP1-coding nucleotide sequence from SAT3 viruses has revealed at least six (I–VI) distinct topotypes. Amongst them, topotypes I–IV occur in southern Africa, whereas topotypes V and VI are unique to Uganda (12, 13). The SAT3 viruses belonging to different topotypes differed by 20% or more in complete nucleotide sequence alignments of the VP1-coding region (12). Studies comparing genetic variation and serological cross-reactivity have shown that SAT1 and SAT2 viruses from different topotypes are generally antigenic poorly related (16, 17). However, similar studies have not yet been undertaken for SAT3 viruses.

Studies focusing exclusively on SAT3 viruses are lacking. Limited studies have been performed to determine the genetic diversity of SAT3 viruses, but these studies were primarily based on partial VP1 sequences. Here, we assessed the intratypic SAT3 genetic variation of the VP1, VP2, VP3, and VP4 capsid proteins and antigenic cross-reactivity within the southern African SAT3 viruses. The analysis of antigenic variation is critical to allow proper vaccine selection or the design of vaccine antigens tailored for specific geographic localities, using reverse genetics.

## Materials and Methods

### Cells and Viruses

Instituto Biologico Renal Suino-2 cells (IB-RS-2) and primary pig kidney (PPK) cells were maintained and propagated in Roswell Park Memorial Institute (RPMI) medium (Sigma-Aldrich) supplemented with 10% (vol/vol) fetal bovine serum (FBS; Delta Bioproducts) and a 1 μg/ml amphotericin B and 0.5 mg/ml gentamycin mixture (Gibco) (18).

Twelve SAT3 viruses, collected from buffalo or cattle during 1990–2010 in southern Africa, were sequenced and used for genetic and antigenic analysis and an additional ten SAT3 P1 sequences available in GenBank were included for the genetic analysis. The viruses form part of the virus databank of the Agricultural Research Council-Onderstepoort Veterinary Research Institute (ARC-OVR), Transboundary Animal Diseases (TAD) Biosafety Level 3 (BSL-3) laboratory (South Africa). The species, which the viruses were isolated from, the country of origin, and year of isolation are summarized in Table 1. The viral isolates were initially passaged on PPK cells, prior to propagation on IB-RS-2 cells and harvested when maximum cytopathic effect (CPE) was observed or after 48 h. All viruses were titrated to determine the tissue culture infectious dose at 50% (TCID_50_). Virus growth medium (VGM) was prepared with RPMI supplemented with 5% (vol/vol) FBS and 1% (vol/vol) antibiotics/antimycotic mixture (Gibco). The SAT3/KNP/10/90, SAT3/SAR/1/06 (topotype I), and SAT3/BOT/6/98 (topotype II) viruses were selected as reference material for the preparation of antisera.

**Table 1:** List of SAT3 viruses used in the current study including species of isolation, passage history, year of isolation, and country of isolation.

**SAT3 virus**	**GenBank accesion numbers**	**Species**	**Passage history**	**Year**	**Country of isolation**	**References**
KNP/10/90	AF286347	Buffalo	PK1RS2	1990	South Africa	This study
KNP/2/03	MK415738	Buffalo	PK1RS2	2003	South Africa	This study
KNP/6/08	MK415735	Buffalo	PK1RS3	2008	South Africa	This study
KNP/14/96	MK415741	Buffalo	PK1RS2	1996	South Africa	This study
KNP/8/02	MK415739	Buffalo	PK1RS1	2002	South Africa	This study
KNP/1/03	MK415737	Buffalo	PK1RS2	2003	South Africa	This study
KNP/1/08	MK415734	Buffalo	PK1RS2	2008	South Africa	(7)
SAR/57/59	AY593850	–	–	1959	South Africa	(19)
SAR/14/01	MK415740	Buffalo	PK1RS2	2001	South Africa	This study
SAR/1/06	MK415736	Buffalo	PK1RS2	2006	South Africa	This study
ZIM/4/81	KX375417	–	–	1981	Zimbabwe	(20)
ZIM/6/91	KM268901	–	–	1991	Zimbabwe	(21)
ZIM/11/94	MK415743	Buffalo	PK1RS5	1994	Zimbabwe	This study
BOT/6/98	MK415742	Buffalo	PK1RS2	1998	Botswana	This study
KEN/11/60	AY593852	–	–	1960	Kenya	(19)
BEC/20/61	AY593851	–	–	1961	Botswana	(19)
BEC/1/65	AY593853	–	–	1965	Botswana	(19)
ZIM/5/91	MK415745	Buffalo	PK1RS5	1991	Zimbabwe	This study
ZAM/5/93	MK415744	Buffalo	PK1RS2	1993	Zambia	This study
ZAM/4/96	DQ009741	–	–	1996	Zambia	(16)
UGA/2/97	DQ009742	–	–	1997	Uganda	(16)
UGA/1/13	KJ820999	–	–	2013	Uganda	(13)

### Virus Titrations

The viral titers were determined in flat-bottomed microtiter plates (Nunc). Briefly, 0.5 log_10_ dilutions of the virus stocks were titrated into 96-well microtitre plates (Nunc), followed by addition of 3 × 10^5^ IB-RS-2 cells per well. Plates were incubated at 37°C with continuous CO_2_ influx. At 72 h post-inoculation the remaining intact cells were stained with 1% (wt/vol) methylene blue in 10% (vol/vol) formalin. The plaques were counted to calculate virus titers, which were expressed as tissue culture infectious dose 50% (TCID_50_) according to the method of Kärber (22).

### RNA Extraction, cDNA Synthesis, PCR Amplification, and Sequencing

Viral RNA was extracted from infected cell culture supernatant using the QIAamp viral RNA mini extraction kit (Qiagen) and used as template for cDNA synthesis (23). First-strand cDNA synthesis was performed using the SuperScript® III First-Strand Synthesis System (Invitrogen) and the genome-specific oligonucleotide 2B (24) following the manufacturer's recommendations. The FMDV ca. 3.0 kb Leader/capsid-coding region was PCR amplified using the Expand High Fidelity PCR system (Roche) and flanking oligonucleotides NCR (5′-TAACAAGCGACACTCGGGATCT-3′) and WDA (5′-GAAGGGCCCAGGGTTGGACTC-3′) (25). Amplicons were purified from an agarose gel with the QIAquick® Gel Extraction Kit (Qiagen). Sequencing of the amplicons was performed using the ABI PRISM™ BigDye Terminator Cycle Sequencing Ready Reaction Kit v3.0 (Perkin Elmer Applied Biosystems) and resolved on an ABI PRISM 3100 Genetic Analyser (Applied Biosystems). The sequences were assembled using Sequencher 5.1 (GeneCodes). The GenBank accession numbers of the capsid-coding sequences are shown in Table 1. The nucleotide sequences were aligned using CLUSTAL_X (26) and phylogenetic trees were constructed using MEGA (27).

### Preparation of Bovine Serum

Convalescent sera were obtained from cattle infected with the respective SAT3 reference viruses (SAT3/KNP/10/90, SAT3/SAR/1/06, and SAT3/BOT/6/98), 28 days post-infection (dpi). Groups of five cattle were inoculated intradermoligually with 1 ml of 10^4^ TCID_50_ per ml of either of the reference viruses. Cattle were housed in the biosafety level 3 stables at the ARC-OVR, TAD. All procedures were approved by the ARC-OVR Animal Ethics Committee (Ethics approval number AEC18.11) according to national animal welfare standards and performed with the permission of the Department of Agriculture, Forestry, and Fisheries (Act 35 of 1984).

Sera collected from each infected cattle were inactivated at 56°C for 30 min. Inactivated sera from the five cattle for each group were pooled and the pooled sera were used in subsequent experiments.

### Virus Neutralization Test

Antigenic cross-reactivity of FMDV against the convalescent animal sera was determined using the virus neutralization test (VNT) according to the World Organization for Animal Health (OIE) Manual of Diagnostic Tests and Vaccines for Terrestrial Animals (28). Briefly, the test serum was diluted 2-fold in VGM using 96-well microtitre (Nunc) plates, starting with a 1/8 dilution, and mixed with a virus suspension containing ~100 TCID_50_ per well. After 1 h of incubation at 37°C, 3 × 10^5^ IB-RS-2 cells were added to each well and incubated for a further 72 h at 37°C in a humid atmosphere containing 5% CO_2_. Cell-only controls were added to each plate and a virus titration control and positive serum control (cells, virus, and positive reference serum) were performed on each day. Plates were analyzed microscopically and colorimetrically for CPE and 50% end-point serum titers were calculated according to the method of Kärber (22). Virus neutralization titers were expressed as the log_10_ of the reciprocal serum dilution that protected the cells in 50% of the inoculated wells. All VNTs were performed at least three times. One-way antigenic relationships (r_1_-value) of the field virus isolates relative to the reference viruses were calculated, and expressed as the ratio between heterologous and homologous serum titer. The criteria of the OIE Manual (28) were applied for interpreting the antigenic relationships. Briefly, r_1_-values between 0 and 0.29 indicated significant antigenic variation from the reference viruses, and values of ≥0.30 demonstrated that the reference and field viruses are sufficiently antigenically similar.

### Virus Purification

BHK-21 cell were seeded, based on cell counts performed using a haemocytometer and tryphan blue staining, into 8 × 750 cm^2^ plastic roller bottles (Corning) to obtain confluent monolayers. Confluent BHK-21 cell monolayers were infected at an multiplicity of infection (MOI) of 5–10 pfu/cell with SAT3/KNP/10/90, SAT3/BOT/6/98, SAT3/SAR/1/06, SAT3/KNP/14/96, or SAT3/SAR/14/01 in Glasgow's Minimal Essential Medium (GMEM) supplemented with 10% (vol/vol) tryptose phosphate broth (TPB), 3% (vol/vol) lactalbumin hydrolysate solution, 1% (vol/vol) FBS, 1% (vol/vol) antibiotic-antimycotic solution, and 25 mM HEPES buffer. Following incubation for 14–16 h at 37°C, the cells were lysed by addition of 10% (vol/vol) Nonidet P-40 and 0.5 M EDTA (pH > 7.4). The virus particles were recovered and concentrated from the lysed cell supernatants as described by Opperman et al. (29). The 146S virus particles were purified on a 10–50% (wt/vol) sucrose density gradient (SDG), prepared in TNE buffer (50 mM Tris [pH 7.5], 150 mM KCl, 10 mM EDTA), as described previously (30). Peak sucrose fractions corresponding to 146S virion particles were pooled and the amount of antigen was calculated (31).

### Single Dilution Avidity ELISA (sd A-ELISA)

The protocol was adapted from Lavoria et al. (32). Briefly, Maxisorp ELISA plates were coated, in duplicate, overnight at 4°C with 200 ng of sucrose density gradient (SDG)-purified virus in 50 mM carbonate/bicarbonate buffer (pH 9.6). The plates were washed with phosphate-buffered saline (PBS) containing 0.05% (vol/vol) Tween-20 (PBS-0.05%T) and blocked at 37°C for 1.5 h with blocking buffer [PBS, 20% (vol/vol) FCS, 0.002% (wt/vol) thimerosal, and 0.1% (wt/vol) phenol red] and washed. The reference sera were diluted 1:40 in blocking buffer, added to the plates and incubated at 37°C for 1 h. The negative control sera consisted of a pool of five negative bovine sera. The plates were washed three times with PBS-0.05%T and then 4 M urea in PBS was added to one plate and PBS was added to the remaining plate. Following incubation at room temperature for 20 min, the plates were washed again before the FMDV-specific antibodies were detected with horseradish peroxidase-labeled anti-bovine conjugate (Sigma-Aldrich), diluted 1:20,000 in blocking buffer (29). The ELISA plates were developed using a substrate/chromogen solution, consisting of 4 mM 3,3′,5,5′-tetramethylbenzidine (Sigma-Aldrich) in substrate buffer (0.1 M citric acid monohydrate, 0.1 M tri-potassium citrate; pH 4.5) and 0.015% (vol/vol) of H_2_O_2_. The color reaction was stopped after 10 min with 1 M H_2_SO_4_ and the optical density (OD) was read at 450 nm using a Labsystems Multiscan Plus photometer. Mean OD values of samples and controls were corrected by subtracting mean blank OD values (cOD). The avidity index (AI) was calculated as described previously (32). Briefly, AI% = (cOD sample with urea/cOD sample without urea) × 100. AI were compared using one-way analysis of variance (ANOVA) and Benferroni's multiple comparison test (33) with a 95% confidence interval (CI) of difference; *p*-value < 0.5 indicated significance binding. The analysis was performed using GraphPad Prism v5.03 for Windows (GraphPad Software, Inc.).

### Statistical Analysis of Gene Sequences and Virus Neutralization Titers

To identify genetic predictors of antigenic variation, amino acid substitutions between reference viruses and test viruses were tested using a model fitted to geometric (log_2_) VN titers, while accounting for phylogenetic relationships and non-antigenic variation in VN titers that can be attributed to day-to-day variability in tests performed on different dates. To prevent false support for substitutions that arise due to the evolutionary process, phylogenetic information was included in the model (17). A phylogenetic tree was reconstructed from aligned capsid nucleotide sequences using PhyML v3.0 (34). The general time reversible model with a proportion of invariant sites and a gamma distribution describing among-site rate variation (GTR + I + Γ_4_) was identified as the best model of nucleotide substitution using jModelTest v2.1.10 (35). Each combination of reference and test virus is separated by a unique combination of branches of the phylogeny. Phylogeny branches separating reference and test viruses were tested as correlating with antigenic change expressed in lower VN titers. In addition, phylogenetic terms associated with changes in immunogenicity were identified (branches). The optimal combination of amino acid position variables and phylogenetic variable was identified using a sparse hierarchical Bayesian model where each variable is associated with parameter estimate and in addition, a binary indicator variable that determines inclusion (1) or exclusion (0) in the model (36). The posterior mean of each indicator variable provides an estimate for the inclusion probability for each variable. Additionally, the model was used to estimate the proportion of all variables tested that should be included in an optimal model. Conditional effect sizes (coefficient estimated for a variable when present in the model, i.e., when associated indicator variable = 1) were mapped to branches of the phylogeny and visualized alongside a heatmap showing VN titers using the *ggtree* R package (37). Separately, a two-dimensional hierarchical clustering of reference and test viruses was performed and visualized as a heatmap.

## Results

### Antigenic Diversity Among SAT3 Viruses in Southern Africa

We applied one-way antigenic relationships (r_1_-values), measured by VNTs, to investigate the antigenic variability of viruses belonging to the SAT3 serotype in southern Africa. SAT3 viruses showed a significant degree of cross-reactivity to the sera of the SAT3 reference viruses (SAT3/SAR/1/06 and SAT3/BOT/6/98) (Table 2). At least 92% (*n* = 11) and 100% (*n* = 12) of the SAT3 viruses showed r_1_-values ≥0.3 to the SAT3/SAR/1/06 (topotype I) and SAT3/BOT/6/98 (topotype II) sera, respectively. However, one of the viruses in topotype I, SAT3/KNP/1/03, had an r_1_-value of <0.2 when tested against SAT3/SAR/1/06, but cross-reacted with SAT3/BOT/6/98 antisera with an r_1_-value of 0.39. Cross-reactivity to the SAT3/KNP/10/90 (topotype I) reference sera indicated that 67% (*n* = 8) of the viruses were neutralized by the sera with an r_1_-value above 0.3. Interestingly, three viruses showed r_1_-values >1.0 against SAT3/BOT/6/98 antisera and four viruses had a similar high cross-reactivity to the SAT3/SAR/1/06 antisera, one of which was as high as 2.24. We then investigated whether the higher neutralization titers of these viruses (KNP/10/90, SAR/14/01, KNP/14/96 and BOT/6/98) were as a result of increased avidity of the antisera to the particular viruses.

**Table 2:** Comparison of the number of variable amino acids in a pairwise alignment of the structural proteins (P1 polypeptide) and r_1_-values between reference viruses and test viruses.

**Strain**	**Topotype^a^**	**SAT3/KNP/10/90**		**SAT3/SAR/01/06**		**SAT3/BOT/06/98**	
		**Variable amino acid^b^**	**r_1_-value^c^**	**Variable amino acid^b^**	**r_1_-value^c^**	**Variable amino acid^b^**	**r_1_-value^c^**
SAT3/KNP/10/90	I	0	1	38	**1.78**	57	**1.28**
SAT3/SAR/14/01	I	40	0.65	44	**1.46**	67	**1.16**
SAT3/ZIM/6/91	I	36	–	37	–	61	–
SAT3/KNP/2/03	I	33	0.39	33	0.6	56	0.45
SAT3/KNP/8/02	I	28	*0.21*	32	0.41	54	0.38
SAT3/SAR/1/06	I	38	*<0.2*	0	1	63	0.87
SAT3/KNP/1/03	I	38	*<0.2*	50	*<0.2*	64	0.39
SAT3/KNP/1/08	I	39	–	37	–	60	–
SAT3/SAR/57/59	I	42	–	41	–	66	–
SAT3/KNP/14/96	I	40	1.09	43	**1.67**	55	**1.32**
SAT3/KNP/6/08	I	36	0.73	42	1	53	1
SAT3/ZIM/4/81	I	34	–	40	–	61	–
SAT3/ZIM/11/94	II	51	0.48	56	1	46	0.83
SAT3/KEN/11/60	II	65	–	77	–	50	–
SAT3/BEC/20/61	II	66	–	78	–	51	–
SAT3/BEC/1/65	II	57	–	61	–	39	–
SAT3/BOT/6/98	II	57	0.6	63	**2.24**	0	1
SAT3/ZIM/5/91	III	59	*0.27*	64	0.54	69	0.64
SAT3/ZAM/4/96	IV	61	–	74	–	62	–
SAT3/ZAM/5/93	IV	56	0.97	70	0.7	58	0.94
SAT3/UGA/2/97	VI	118	–	119	–	113	–
SAT3/UGA/1/13	VI	122	–	128	–	124	–

a *The topotypes classification is based on the VP1 phylogeny proposed by Vosloo et al. (38) and Bastos et al. (12)*.

b *Pairwise alignment was performed for the complete P1 polypeptide of 741 amino acids*.

c *r_1_-values higher than 1 are indicated in bold and those values lower than 0.3 in italics. The homologous r_1_-values are highlighted in light gray*.

*“–” VNTs were not performed and sequences of these viruses were retrieved from GenBank*.

**Figure 1:**
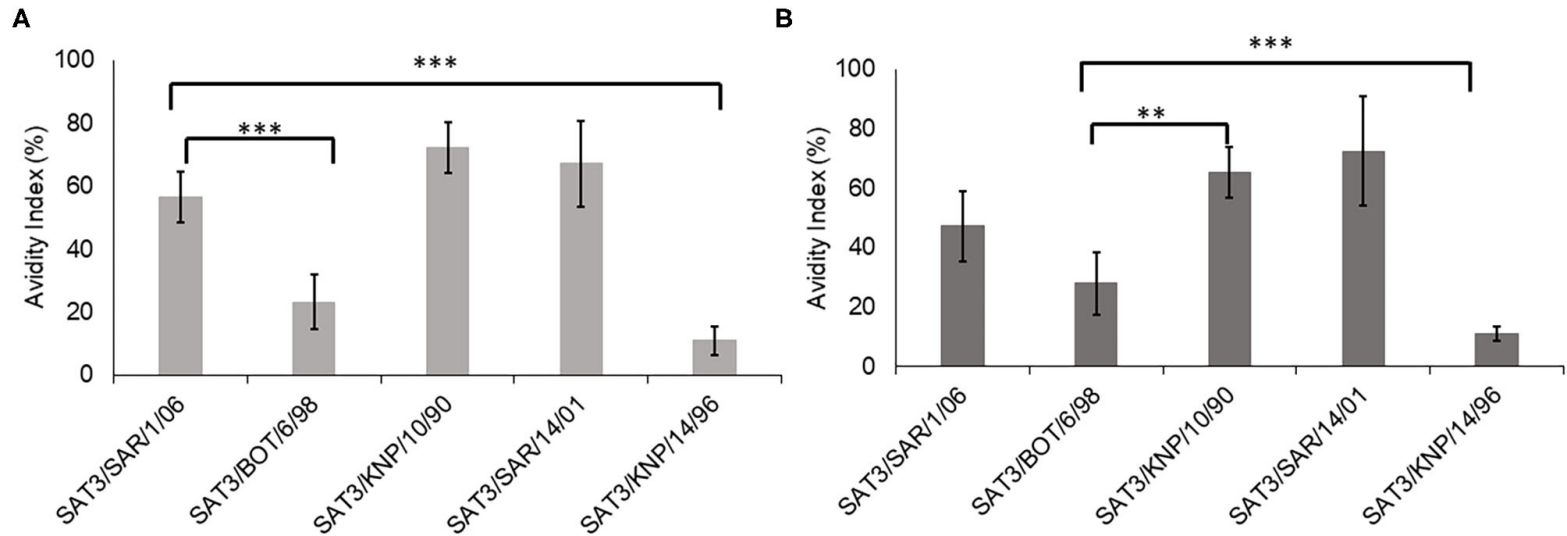
Avidity index of the SDG-purified SAT3 viruses SAT3/SAR/1/06, SAT3/BOT/6/98, SAT3/KNP/10/90, SAT3/SAR/14/01, and SAT3/KNP/14/96, to the bovine antisera raised against SAT3/SAR/1/06 **(A)** and SAT3/BOT/6/98 **(B)** are indicated. The data are means ± SD of quadruplicate experiments. **p < 0.01, ***p < 0.001 at 95% CI.

The avidity index of the SAT3/BOT/6/98 and SAT3/SAR/1/06 bovine antisera against the SAT3 viruses with r_1_-values >1.0 (SAT3/KNP/10/90, SAT3/SAR/14/01, SAT3/KNP/14/96), and the homologous viruses is shown in Figure 1. The avidity index of the SAT3/KNP/10/90 (AI = 72%) and SAT3/SAR/14/01 (AI = 67%) viruses to the SAT3/SAR/1/06 antisera was higher than the avidity to the homologous virus (AI = 56%), albeit statistically insignificant (*p* > 0.05) (Figure 1A). Avidity values of <25% were observed for SAT3/BOT/6/98 and SAT3/KNP/14/96 viruses to the SAT3/SAR/1/06 antisera. In contrast, antibodies in SAT3/BOT/6/98 antisera bound with high avidity to the SAT3/KNP/10/90 (AI = 65%; *p* < 0.01), SAT3/SAR/14/01 (AI = 72%; *p* < 0.001), and SAT3/SAR/1/06 (AI = 47%; *p* > 0.05) viruses, while the avidity against the SAT3/KNP/14/96 (AI = 11%) and the homologous virus, SAT3/BOT/6/98 (AI = 28%), was lower (Figure 1B).

### Genetic Variation in the Capsid Proteins of SAT3 Viruses

The intratypic nucleotide variation of the SAT3 P1 region was calculated to be 45.6% (*n* = 22) and is comparable to the intratypic variation reported for SAT1 (47.3%; *n* = 20) and SAT2 (48.9%; *n* = 23) viruses, but higher than types A (42.5%; *n* = 50) and O (38.2%; *n* = 41) (16, 19). The nucleotide and amino acid variation in a complete alignment of the SAT3 capsid proteins and coding region is summarized in Table 3. With the exception of SAT3/KNP/10/90, the P1 region of SAT3 viruses was 2,220 nucleotides in length and encodes 740 amino acids representing the four structural proteins. The VP1-coding region of SAT3/KNP/10/90 contains a three-nucleotide-insertion between nucleotides 252 and 253, which translates to an additional amino acid (lysine, K) in the βD-βE loop of the VP1 protein. Overall, in the capsid coding region, a total of 1,015 (45.7%) nucleotide positions were variant. The majority of the mutations in the P1 region (36.5%) were synonymous; however, at least 45% of the nucleotide substitutions in the VP1-coding region resulted in amino acid changes in the complete alignment.

**Table 3:** Variation within the nucleotide and amino acid sequences of the P1 coding region and deduced polyprotein in a complete alignment to each of the SAT3 reference viruses.

**Genome region**	**No. nucleotide positions aligned**	**No. variant nucleotides**	**Variant nucleotides (%)**	**No. amino acid positions aligned**	**No. variant amino acids**	**Variant amino acids (%)**
VP4 (1A)	258	84	32.5	86	1	1.2
VP2 (1B)	651	276	42.4	217	54	24.8
VP3 (1C)	663	272	41.0	221	52	23.5
VP1 (1D)	651/4	377	57.9	217/8	98	45.2
P1	2220/1	1015	45.6	740/1	205	27.7

**Figure 2:**
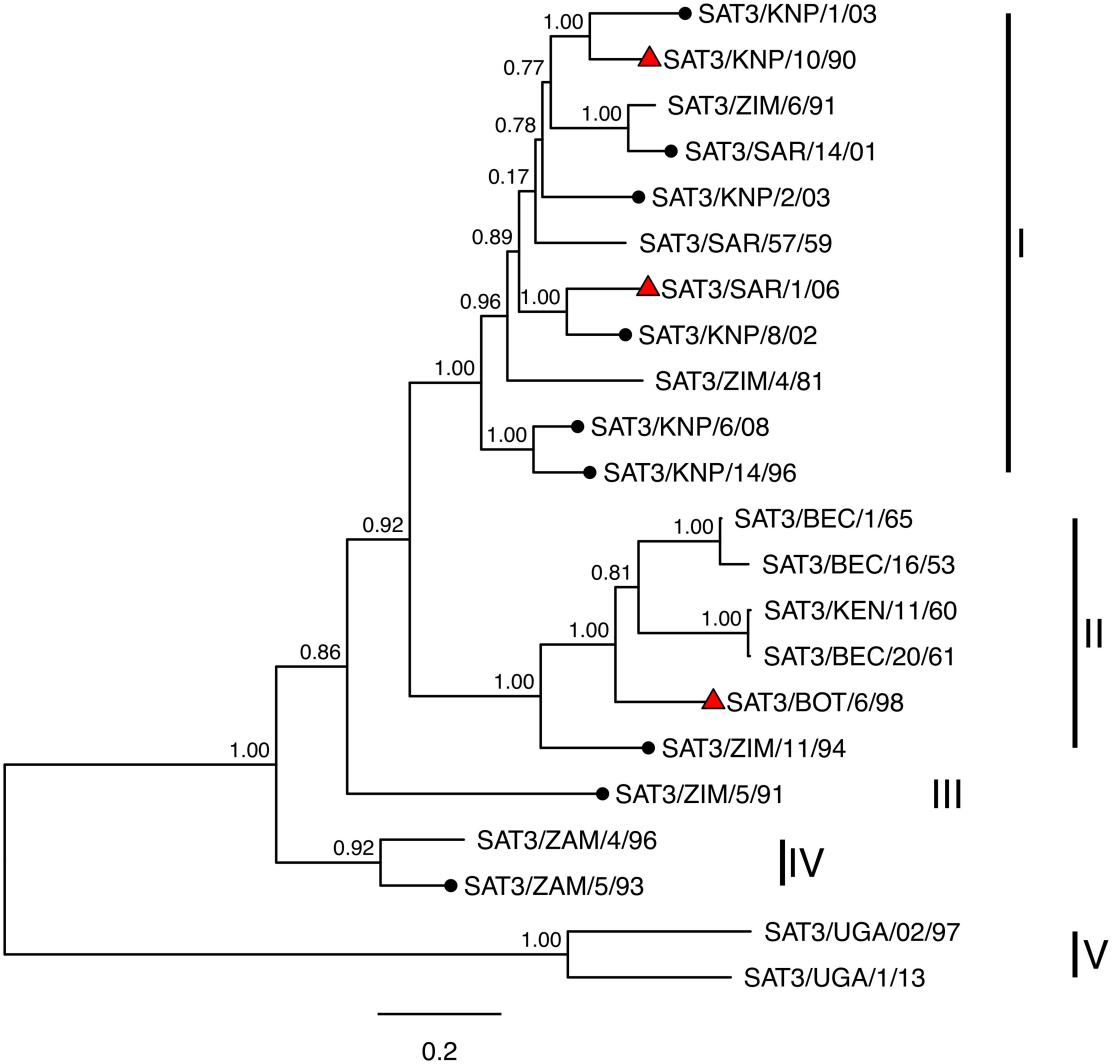
Maximum likelihood phylogenetic tree constructed from aligned capsid (P1) nucleotide sequences, with mid-point root. Clades of the phylogeny corresponding to topotypes are labeled. Viruses tested as antigen in virus neutralization (VN) assays are marked with black circles and reference viruses also used to generate antiserum for VN assays are marked with red triangles. Internal nodes are labeled with bootstrap values and branch lengths indicate the estimated number of nucleotide substitutions per site.

A maximum phylogenetic tree constructed from this alignment with topotypes and the positions of viruses further investigated using virus neutralization assays is shown in Figure 2. Phylogenetic resolution of capsid protein sequences of the SAT3 viruses confirmed five of the six topotypes, each with its unique geographic distribution. Topotype I included viruses from South Africa and southern Zimbabwe, topotype II encompassed viruses from Botswana and western Zimbabwe, and topotype IV viruses from Zambia.

A pairwise alignment of the capsid proteins of the SAT3 viruses with the corresponding proteins of each reference virus displayed variation in 28–70 of the amino acid positions, with most variation in the pairwise alignments with SAT3/BOT/6/98 (46–69 variable residues) (Table 2). No clear correlation was observed between the number of variable residues and r_1_-values to each of the reference viruses (Table 2).

In a complete alignment of the structural proteins, three regions of notable variability (amino acid entropy >1) were observed in the VP2 protein at amino acid positions 92–101 (βC-βD loop), 128–138 (βE-βF loop), and 208–217. In the VP3 protein, 23.5% variable amino acid positions were observed and residues with high entropy (>1) were positioned on the surface-exposed βE-βF loop at 131, 135, and 139 and in the C-terminus at residues 219–220. However, several regions with hypervariability were identified throughout the VP1 protein including: (i) N-terminal residues 7–16; (ii) the linear amino acid region that correlates with a T-cell epitope region in serotype O (39), also in the N-terminus (aa 21–26); (iii) a region in the βB-βC loop (aa 44–55) correlating with O1BFS antigenic site 3 (40); (iv) βD-βE loop (aa 79–91); (v) βF-βG loop (aa 109–116); (vi) residues 137–146 and 149–163 of the βG-βH loop; (vii) residues 175–185 and lastly, (viii) the C-terminus (aa 196–206 and aa 207–216).

### Predicting Antigenic Substitutions in the Outer Capsid Proteins of SAT3 Viruses

**Figure 3:**
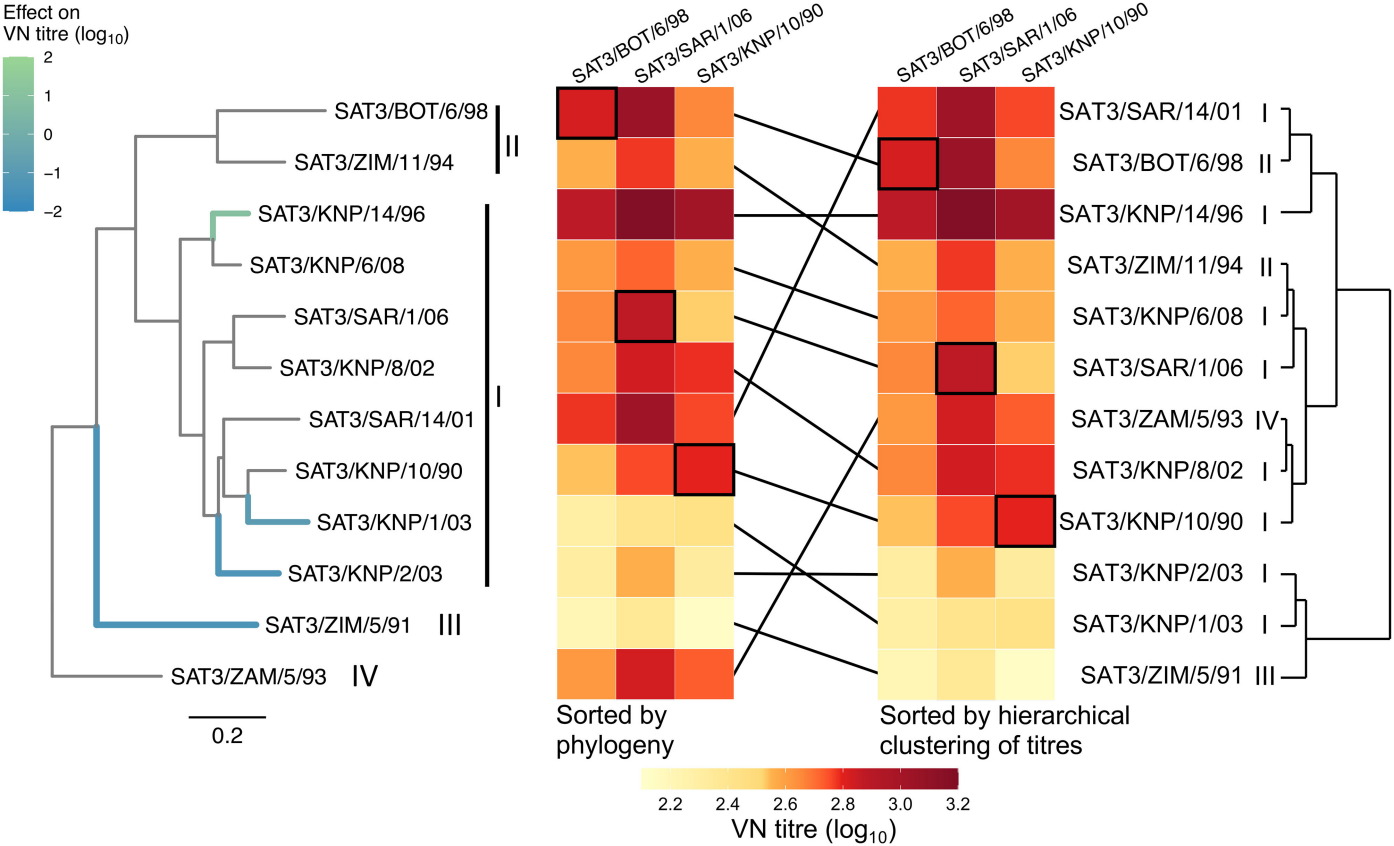
Heatmaps of virus neutralization titers (VN) ordered by phylogeny and by hierarchical clustering. The two heatmaps show the same VN titers (log10) for 12 viruses (rows) and reference antisera (columns) raised to reference viruses SAT3/BOT/6/98, SAT3/SAR/1/06, and SAT3/KNP/10/90. To the left, rows of the heatmap are ordered according to the phylogenetic tree constructed from capsid nucleotide sequences (far left). To the right, rows of the heatmap are ordered according to a hierarchical clustering algorithm applied to VN titers—a dendogram generated by this algorithm is shown (far right). In both heatmaps, black framing is used to highlight homologous titers. Highlighted branches of the phylogeny were associated with variation in VN titers using a sparse hierarchical Bayesian model. Branch color indicates the average effect on titers: green indicates a branch where amino acid substitutions tended to lead to an increase in the VN titer to the three reference sera pools, while blue branches correlated with decreases in VN titers. No internal branches of the phylogeny tended to correlate with variation in VN titers. Topotype nomenclature appears next to clades of the phylogeny and alongside each virus name associated with the hierarchical clustering dendogram.

Next, we explored the genetic basis of variation expressed in VN titers. In Figure 3, two heatmaps show the same VN titers (log10) for 12 viruses (rows) and reference antisera (columns) raised to three reference viruses organized in two ways: firstly, where test viruses (rows) are sorted according to the phylogeny and secondly where test viruses (rows) are sorted according to a hierarchical clustering of the VN titers. The hierarchical clustering analysis, also expressed in the dendograms to the right of the heatmaps, indicated that viruses of the same topotype did not consistently cluster together on the basis of cross-reactivity data.

To probe the relationship between VN titers and genetic differences in greater detail, a sparse hierarchical Bayesian model was used to test whether substitutions at each non-conserved amino acid residue, within the VP1, VP2, and VP3 proteins, were predictors of reduced antigenic cross-reactivity. Residues would be selected if substitutions between test and reference virus tended to correlate with lower VN titers. The model also accounted for other sources of variation in measured VN titers (Supplementary Figure 1). When compared with the mean titers recorded for each virus and reference virus combination, the mean difference to these for individual recorded titers was 0.25 log_10_ titer (maximum 0.89). Some of the variation in recorded titers was attributed to day-to-day variability in the assay (Supplementary Figure 1B). The average residual difference between measured and fitted titers, after accounting for day-to-day variability was reduced to 0.15 log_10_ titer (maximum 0.83).

Variable representing amino acid substitutions were tested alongside terms representing branches of the phylogeny that could also identify branches leading to individual viruses or groups of viruses that tended to have higher VN titers, perhaps as a result of differences in avidity for the cellular receptor. Four well-supported branches, to which variation in VN titers mapped, are shown in the phylogenetic tree in Figure 3. Each branch effect is caused by the combined effect of one or more residue changes that significantly affect cross-reactivity between reference and test viruses. Each of the four identified branches were terminal branches leading to a single virus, three correlated with low VN titers and one branch with higher VN titers. The terminal branch for SAT3/KNP/14/96 significantly accounts (inclusion probability = 0.97) for an increase in antigenic cross-reactivity to all three reference sera pools. The increase in cross-reactivity reflected as high VN titers regardless of antisera used and was not due to a higher virus titer (4.7 ± 0.2 log_10_/ml) or increased avidity (AI = 13.95). Three branches in the phylogenetic tree significantly accounted for a reduction in antigenic cross-reactivity against all three reference sera pools. These branches could indicate that the viruses are antigenically distinct, or that they have low VN titers as a result of increased avidity for the cellular receptor. One highlighted branch caused a partitioning of a single topotype (III) from the rest of the tree (SAT3/ZIM/5/91). The virus SAT3/KNP/1/03, although genetically similar to SAT3/KNP/10/90 (38 aa differences in the capsid proteins), is antigenically distinct from the SAT3/KNP/10/90 and SAT3/SAR/1/06 reference viruses (r_1_-value <0.3). Similarly, the separation of SAT3/KNP/2/03 from the other topotype I viruses was associated with a decrease in cross-reactivity to the reference sera pools. From our data, the topotype IV virus SAT3/ZAM/5/93 does not seem to be antigenically different from the topotype I and II viruses.

The three branches in the phylogeny in Figure 3 identified as correlating with reduced VN titers lead to single viruses that are potentially antigenically distinct due to amino acid residue substitutions in the capsid protein. Amino acid substitutions mapping to each of these branches were identified. The branch leading to the virus SAT3/ZIM/5/91 correlated with 13 residue substitutions in the outer capsid proteins, while the branch leading to SAT3/KNP/1/03 correlated with substitutions at five residue positions, therefore there were several candidate substitutions in these two instances. Only two amino acid substitutions, VP1 L83Q and C164R, mapped to the terminal branch separating SAT3/KNP/2/03 from the rest of the tree; in fact, VP1 83L and 164C are conserved across each of the other 11 viruses in the dataset. Therefore, the substitutions VP1 L83Q and C164R are plausible candidates for causing a reduction in antigenic cross-reactivity. Of these two residues, VP1 164 aligns to a residue that is part of a known epitope in serotype O (17).

**Figure 4:**
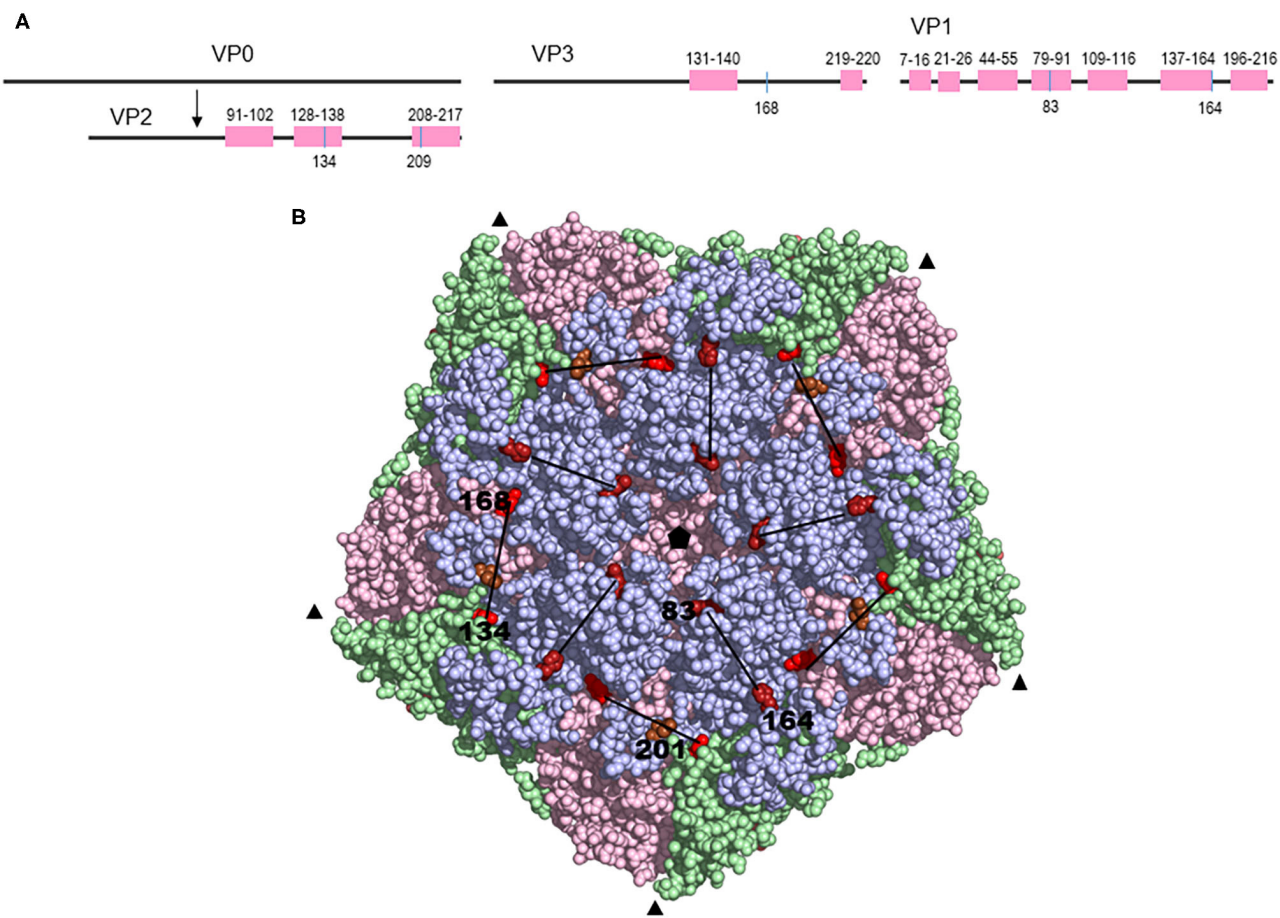
**(A)** Schematic diagram of the capsid proteins showing the amino acid variation in a complete alignment and the relative positions of amino acid substitutions explaining a decrease in the VN titers. **(B)** A model of the FMDV SAT3 pentamer showing the amino acid residues that correspond to branch formation in a phylogenetic tree and a decline in cross-reactivity in VNTs. The inferred 3-D structure were generated using the SAT1 virus (protein data bank ID: 2WZR) as a template and the structural model rendered by Pymol v 1.8 (DeLano Scientific LLC). The capsid proteins VP1, VP2, and VP3 are shown in blue, green, and pink, respectively, while the exposed amino acid variants are indicated by red spheres. The 3-fold axis is depicted by the black triangles. The pore, located at the 5-fold axis of the capsid (black pentagon), is shown in the middle of the structure. The black lines connect the residues that changed simultaneously.

Across the phylogeny, three other terms representing amino acid substitutions were identified as correlating with reduced VN titers (Table 4) (model selection indicated terms with posterior inclusion probability >0.25 to have a reasonable level of support). The first of these terms with greatest support (inclusion probability = 0.88) represented simultaneous substitutions at VP2 residue 134 [Lys (10), Gln (1), Thr (1)] and VP3 168 [Phe (10), Tyr (2)], which only substituted together in this dataset and therefore could not be distinguished. The positions in the phylogeny where these residues were both substituted were terminal branches leading to viruses SAT3/KNP/1/03 (VP2 K134Q and VP3 F168Y) and SAT3/ZIM/5/91 (VP2 K134Q and VP3 F168Y), both of which had low titers against each of the three antisera used. Of these two residues, the VP2 residue 134 has been identified as being part of an epitope for serotype O and SAT2 viruses (17, 29, 40). Finally, genetic terms associated with VP1 residue 201 [Thr (8), Val (2), Ala (1), Arg (1)], which forms part of the VP1 C-terminus, and VP2 residue 209 [Tyr (9), Phe (2), His (1)] were also identified as potentially antigenically important with substitution, though with reduced support. The location of the latter six residues can be resolved on the predicted structure of serotype SAT3 capsid and is shown in Figure 4.

**Table 4:** Amino acid positions in the SAT3 capsid proteins with substitutions explaining a decrease in the VN titers.

Capsid protein and amino acid position in the SAT3 alignment^*^	**Serotype(s) where residue is antigenic**	**Antigenically distinct amino acids**	**Inclusion probability**	**Impact of substitutions on cross-reactivity (log_10_ VN titer)**
VP1 83/VP1 164	None/O^3^	L-Q, C-R	0.88	−1.2
VP2 134/VP3 168	SAT2, O^1,2^/None	K-Q-T, F-Y	0.42	−0.6
VP1 201		T-V-A-R	0.32	−0.22
VP2 209		Y-F-H	0.30	−0.37

*Substitutions at each amino acid position (or combination of positions sharing the same pattern of substitution across viruses in the VN dataset) was tested in a Bayesian model with an indicator variable determining inclusion (1) or exclusion (0) from the model and a coefficient or effect size. The inclusion probability represents the posterior mean value of the indicator variable and the level of support. Model fitting indicated inclusion probabilities above 0.25 have been reasonably well-supported. For each position(s), the conditional effect size is the estimated average impact on VN titers when substitution(s) between test and reference viruses are present*.

* *Where more than one amino acid appears in a row, this indicates the pattern of substitution at these residues to be identical in the dataset*.

## Discussion

The present study confirms the close antigenic relationship between SAT3 viruses in southern Africa using *in vitro* cross-reactivity studies. We then used the antigenic variability data and corresponding genetic and structural data to predict naturally occurring amino acid positions that correlated with antigenic changes. Knowledge of the molecular mechanisms of antigenic evolution are essential to implement systematic approaches to predict protection offered by reference vaccine viruses during prophylactic vaccination in endemic regions or emergency vaccination during an outbreak.

Of the three SAT serotype FMD viruses that occur in southern Africa, SAT3 has the most restricted distribution and outbreaks in livestock are only observed sporadically every 8–15 years (12). A comparison of the genetic diversity within the VP1 coding region of SAT3 viruses, collected between 1965 and 1999 in southern Africa and South-western Uganda, divided the SAT3 viruses into six topotypes (12). Findings from our study, using the complete P1 capsid-coding sequences of SAT3 viruses recovered between 1990 and 2008, substantiated the topotype definitions for SAT3 viruses in southern Africa. Viruses recovered from buffalo in the Kruger National Park, South Africa, and southern Zimbabwe clustered together based on the capsid-coding sequences. Topotype I lineage viruses in South Africa are maintained in buffalo from the Kruger National Park with an incursion every 8–15 years to cattle neighboring this endemic area. Topotype II viruses include viruses from Botswana and western Zimbabwe, while virus isolates from Zambia clustered separately in the phylogenetic tree, defined as topotype IV. The single isolate from northern Zimbabwe was genetically distinct and correlated to topotype III, as described by Bastos et al. (12).

The *in vitro* cross-reactivity analysis of SAT3 viruses was notably high, i.e., 67, 92, and 100% of the SAT3 viruses reacted strongly (r_1_-values ≥0.3) to the SAT3/KNP/10/90, SAT3/SAR/1/06, and SAT3/BOT/6/98 reference antisera. The implication is that, in a case of a cattle outbreak, vaccines consisting of any one of the three reference viruses will provide sufficient protection. Moreover, some of the SAT3 viruses reacted stronger to the reference sera than with the homologous virus (r_1_-value >1). Particularly, the SAT3/SAR/1/06 and SAT3/BOT/6/98 antisera were highly cross-reactive to the test viruses as indicated by r_1_-values >1. Similar results where heterologous cross-reactivity was higher than homologous reactivity have been documented with serotype A FMDV (17, 41). These findings indicate (i) similarities in shared epitopes between the reference and the field viruses, (ii) the reference viruses elicited broadly reactive antibodies in cattle, or (iii) antibodies with high avidity to SAT3 viruses were present. In an attempt to further investigate factors influencing this cross-reactivity, an avidity ELISA was performed to assess and characterize this high heterologous cross-reactivity (32, 42). An increased avidity of SAT3/BOT/6/98 antisera in binding to heterologous viruses (i.e., SAT3/KNP/10/90 and SAT3/SAR/14/01) could explain the higher neutralization titers observed for these viruses. Although higher avidity indexes have been linked to high neutralization titer, this is not always the case. Other factors, such as antibody class or IgG isotype, may also play a role.

The high amino acid variation of the VP1 protein (45% variable residue positions), compared to the other capsid proteins, indicates that VP1 is likely to be under immunologic pressure. Genetic changes and selection of antigenic variants are generally accepted to occur in persistently infected wildlife (8, 43, 44). The majority of variable residues are limited to particular surface-exposed structural loops and changes elsewhere may be under stringent structural and selective constraints (45). The fact that most of the SAT3 capsid amino acid positions with high entropy were identified in the VP1 protein emphasizes that this protein has a major immunogenic role and it also modulates the antigenic variability of the virus. Previous crystallographic studies and structure-based epitope predictions revealed that VP1 is important to interact with antibodies, especially the βG-βH loop and residues toward the 5-fold axis of the capsid (46, 47). The immunological role of an additional K residue within the βD-βE loop of the VP1 protein of one isolate is unknown. Although less variation was identified in the VP2 and VP3 proteins, these proteins still play an important role in antigenic variation of FMDV. A conformational epitope comprising of residues from the VP2 and VP3 capsid proteins and spanning the 3-fold axis, was also present (47–49). This emphasizes that cross-reactivity is influenced by main, variable capsid amino acid residues and may be affected more by residue interactions rather than residue changes (48, 50–52).

We identified substitutions with a profound effect on antigenic variation that were likely associated with immune evasion. Variation at two residue positions in the VP1 protein, residues 83 and 169, were associated with reduced titers against SAT3/KNP/10/90 and SAT3/SAR/1/06 antisera. The VP1 residues 83 and 169 are located at opposite sides of an elevated plateau on the capsid surface, with residue 83 forming an exposed cluster around the 5-fold axis and residue 169 located at the C-terminal base of the VP1 βG-βH loop. The VP1 residue 83 of SAT2 viruses has been found to be accessible to interact with glycosaminoglycan (18), confirming its accessibility to interact with cellular receptors. Similarly, residues 134 in VP2 and 168 in VP3 together, were associated with an antigenic effect for SAT3/KNP/1/03 and SAT3/ZIM/5/91. It is reasonable to hypothesize that the two residues together function as a conformational epitope, however, the same variation in VN titer is equally well-explained by VP2 K134Q/T substitution. The VP2 residue 134 has been described as an antigenic site for serotypes O and SAT2, and is located on a surface exposed structural loop and is structurally more favorable to contribute to variation in antigenicity (48, 49). Residue 168 in VP3 has not been described to play a role in antigenicity before. Both residues are located in a shallow, structural depression, located at the junction between the three major capsid proteins VP2, VP3, and VP1 (Figure 4). Two other residues have also been associated with antigenic variation in SAT3 viruses, one in the C-terminal end of VP2 and the other located on the C-terminus of VP1. Only the VP1 C-terminus residue corresponds to a described antigenic site in serotype O (40, 48).

Amino acids that are important for the antigenicity of SAT1 viruses have been identified at positions 135 or 71 or 76 of VP3; 72 of VP2 and 181 of VP1; and 111 of VP1 using MAb resistant (mar) mutants (49). Similarly, residues 72 or 79 of VP2; 158 of VP1; and 154 or 158 in the βG-βH loop of VP1 of SAT2 viruses have been shown to interact with MAbs or affect the antigenicity of the virus (29, 49). At least five neutralizing antigenic sites, involving the three outer-capsid proteins, have been identified for serotype O viruses. The most prominent surface exposed structure, the βG-βH loop of VP1, and the C-terminus of VP1 have been shown to contribute to antigenic site 1 of serotype O viruses, with critical residues at position 144, 148, 154, and 208 (53–56). Amino acid residues at positions 70–73, 75, 77, and 131 of VP2, 56 and 58 of VP3, and 43 and 44 of VP1 contributes to the remaining antigenic site for serotype O (56). Mar-mutants identified three antigenic sites within the VP1, VP2, and VP3 proteins for serotype A viruses A10, A12, and A22 with residue positions 148, 149, 152, 153, 168, and 205 within VP1 important for antigenicity (57–60). Here, for the first time, we have mapped four unique amino acid regions associated with antigenic changes in SAT3 viruses. In two of these regions two amino acid residues changed together to affect the antigenicity of the virus, i.e., residues 83 and 169 of VP1 and residues 134 in VP2 and 168 in VP3.

We have successfully used phenotypic data, combined with genotypic and structural information in our mathematical models to delineate antigenic sites for SAT3 viruses. The analysis of antigenic differences in outbreak viruses is critical to allow proper vaccine selection for effective control or the design of vaccine antigens tailored for specific geographic localities, using reverse genetics. This work could be further validated using a reverse genetics approach to immune-dampen specific residues to identify its antigenic significance. We anticipate that identifying unique residues associated with a change in cross-reactivity will contribute to improved vaccine development and assessment.

## Data Availability Statement

The datasets presented in this study can be found in online repositories. The names of the repository/repositories and accession number(s) can be found in the article/Supplementary Material.

## Ethics Statement

The animal study was reviewed and approved by Agricultural Research Council, Onderstepoort Veterinary Research, Animal Ethics Committee.

## Author Contributions

WH, JT, RR, and FM conceived and designed the experiments. LM, WH, LR, and PO performed the experiments. RR and FM contributed the materials/analysis tools. LM, WH, LR, PO, JT, RR, and FM wrote the paper. All authors contributed to the article and approved the submitted version.

## Conflict of Interest

The authors declare that the research was conducted in the absence of any commercial or financial relationships that could be construed as a potential conflict of interest. The reviewer DK declared a past collaboration with the author RR to the handling editor.

## Supplementary Material

Supplementary Figure 1 Variability in virus neutralization titers (log_10_) for 12 viruses tested using antisera raised to reference strains SAT3/BOT/6/98, SAT3/SAR/1/06, and SAT3/KNP/10/90 (*N* = 198). **(A)** Histogram of absolute differences in measured titers and the mean log_10_ titer recorded for each virus and reference strain combination. **(B)** Violin plot showing posterior model estimates of the variation in VN titers that can be attributed to variability between experiments carried out on 15 different days. Each violin represents 1,600 values sampled from eight independent MCMC chains. Black horizontal lines represent median values. **(C)** Histogram showing residuals from a model fitted to VN titers—each residual is the absolute difference between a measured titer adjusted for day-to-day variation and the fitted, underlying titer for the particular virus and reference strain combination. **(D)** Scatterplot showing measured VN titers (black circles) and those same VN titers adjusted to account for day-to-day variability (red crosses) plotted against the fitted, underlying titer for a particular virus and reference strain combination.
