# Supplementary figures and images for: Genetic Basis of Antigenic Variation of SAT3 Foot-And-Mouth Disease Viruses in Southern Africa
Source: Front Vet Sci. 2020 Sep 8;7:568. doi: 10.3389/fvets.2020.00568 (PMC7506032; doi:10.3389/fvets.2020.00568)

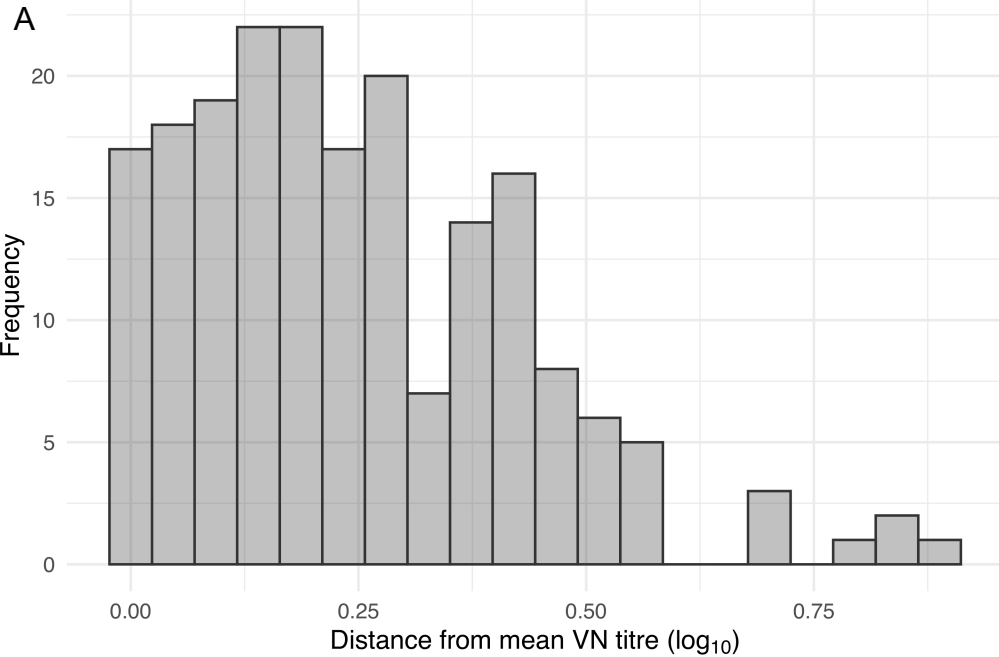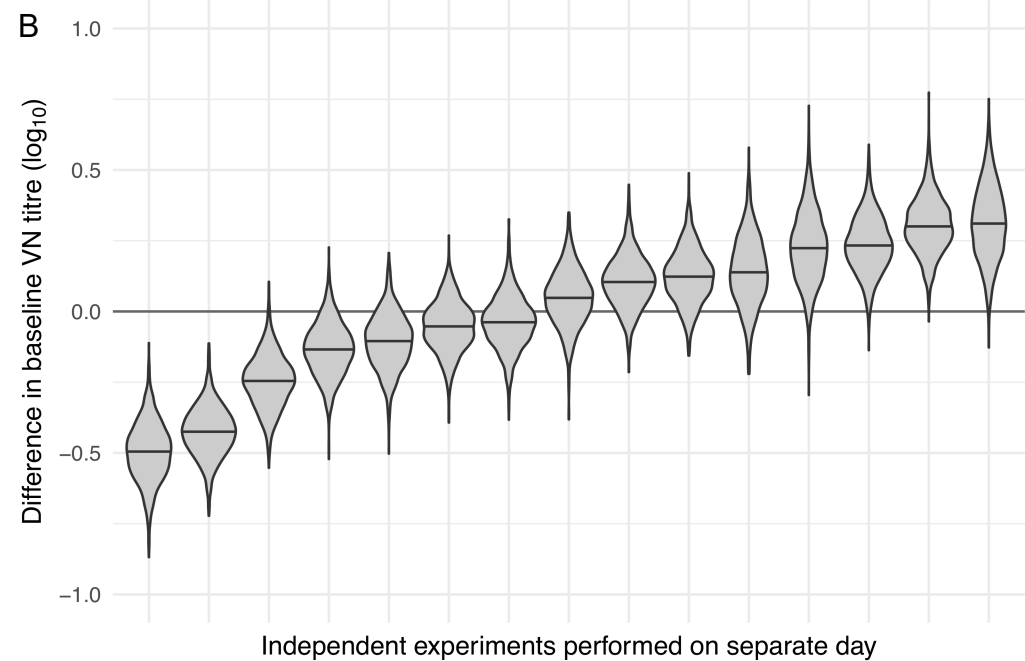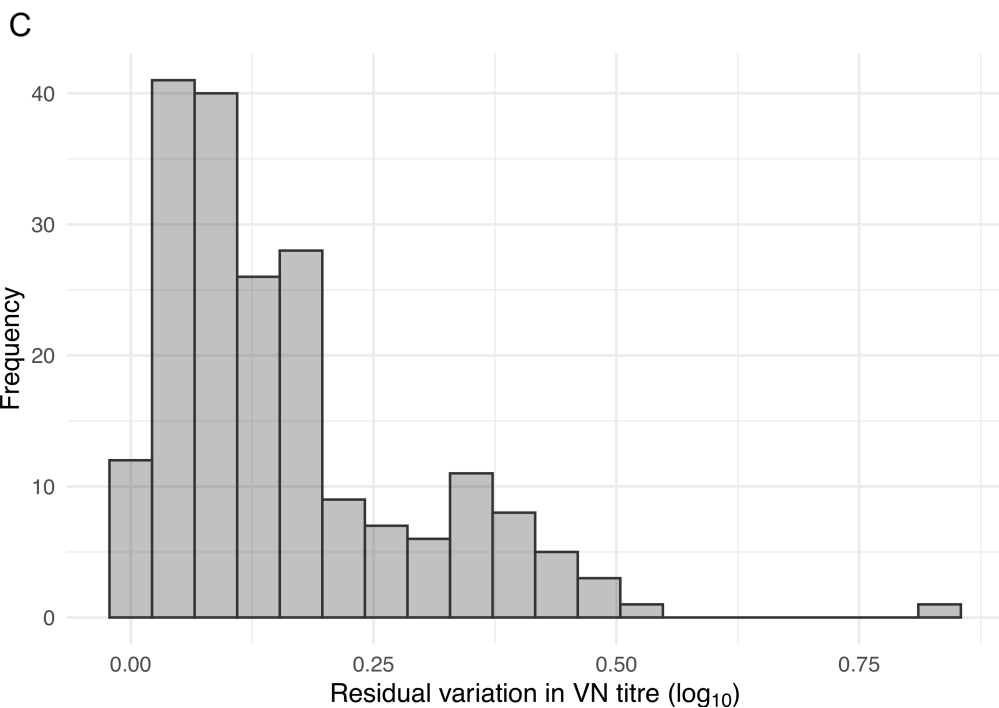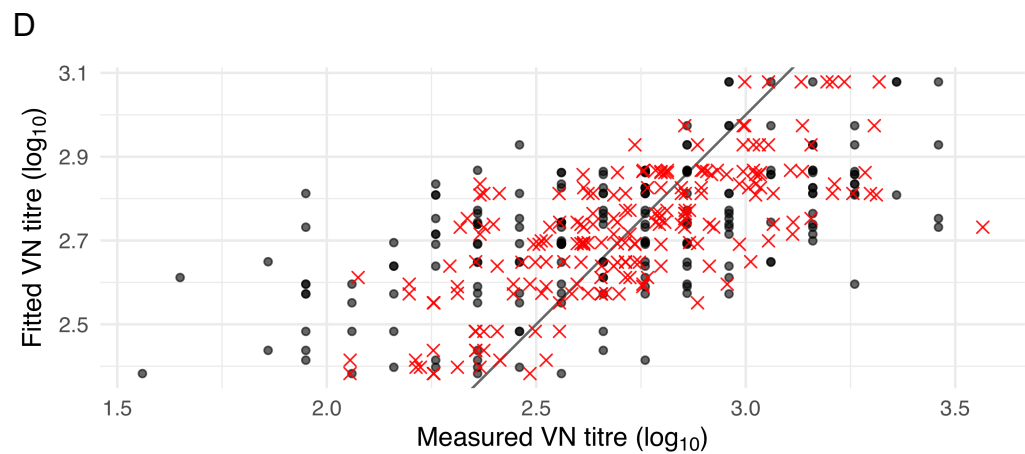

Supplement: Supplementary Figure 1 — Variability in virus neutralization titers (log10) for 12 viruses tested using antisera raised to reference strains SAT3/BOT/6/98, SAT3/SAR/1/06, and SAT3/KNP/10/90 (N = 198). (A) Histogram of absolute differences in measured titers and the mean log10 titer recorded for each virus and reference strain combination. (B) Violin plot showing posterior model estimates of the variation in VN titers that can be attributed to variability between experiments carried out on 15 different days. Each violin represents 1,600 values sampled from eight independent MCMC chains. Black horizontal lines represent median values. (C) Histogram showing residuals from a model fitted to VN titers—each residual is the absolute difference between a measured titer adjusted for day-to-day variation and the fitted, underlying titer for the particular virus and reference strain combination. (D) Scatterplot showing measured VN titers (black circles) and those same VN titers adjusted to account for day-to-day variability (red crosses) plotted against the fitted, underlying titer for a particular virus and reference strain combination. [file Image_1.pdf]
